# Between Lake Baikal and the Baltic Sea: genomic history of the gateway to Europe

**DOI:** 10.1186/s12863-017-0578-3

**Published:** 2017-12-28

**Authors:** Petr Triska, Nikolay Chekanov, Vadim Stepanov, Elza K. Khusnutdinova, Ganesh Prasad Arun Kumar, Vita Akhmetova, Konstantin Babalyan, Eugenia Boulygina, Vladimir Kharkov, Marina Gubina, Irina Khidiyatova, Irina Khitrinskaya, Ekaterina E. Khrameeva, Rita Khusainova, Natalia Konovalova, Sergey Litvinov, Andrey Marusin, Alexandr M. Mazur, Valery Puzyrev, Dinara Ivanoshchuk, Maria Spiridonova, Anton Teslyuk, Svetlana Tsygankova, Martin Triska, Natalya Trofimova, Edward Vajda, Oleg Balanovsky, Ancha Baranova, Konstantin Skryabin, Tatiana V. Tatarinova, Egor Prokhortchouk

**Affiliations:** 1 Children’s Hospital Los Angeles, Los Angeles, CA USA; 2 Federal State Institution “Federal Research Centre «Fundamentals of Biotechnology» of the Russian Academy of Sciences”, Moscow, Russia; 3 “Genoanalytica” CJSC, Moscow, Russia; 4 Institute of Medical Genetics, Tomsk National Medical Research Center, Russian Academy of Sciences, Siberian Branch, Tomsk, Russia; 5 Institute of Biochemistry and Genetics, Russian Academy of Sciences, Ufa Scientific Centre of Russian Academy of Sciences, Ufa, Russia; 6 Bashkir State University, Ufa, Russia; 7 School of Chemical and Biotechnology, SASTRA University, Tanjore, India; 8 Moscow Institute of Physics and Technology, Department of Molecular and Bio-Physics, Moscow, Russia; 9 Russian Scientific Centre “Kurchatov Institute”, Moscow, Russia; 10 Institute of Cytology and Genetics, Russian Academy of Sciences, Siberian Branch, Novosibirsk, Russia; 11 Skolkovo Institute of Science and Technology, Skolkovo Innovation Center, Moscow, Russia; 12 Tyumen State Medical Academy, Tyumen, Russia; 13 Department of Modern and Classical Languages, Western Washington University, Bellingham, WA USA; 14 Research Centre for Medical Genetics, Moscow, Russia; 15 Vavilov Institute of General Genetics, Moscow, Russia; 16 School of Systems Biology, George Mason University, Fairfax, VA USA; 17 Atlas Biomed Group, Moscow, Russia; 18 Department of Biology, Lomonosov Moscow State University, Moscow, Russia; 19 Department of Biology, University of La Verne, La Verne, CA USA; 20 A. A. Kharkevich Institute for Information Transmission Problems, Russian Academy of Sciences, Moscow, Russia

**Keywords:** Population genetics, Siberia, Eastern Europe, IBD, Admixture, Biogeography

## Abstract

**Background:**

The history of human populations occupying the plains and mountain ridges separating Europe from Asia has been eventful, as these natural obstacles were crossed westward by multiple waves of Turkic and Uralic-speaking migrants as well as eastward by Europeans. Unfortunately, the material records of history of this region are not dense enough to reconstruct details of population history. These considerations stimulate growing interest to obtain a genetic picture of the demographic history of migrations and admixture in Northern Eurasia.

**Results:**

We genotyped and analyzed 1076 individuals from 30 populations with geographical coverage spanning from Baltic Sea to Baikal Lake. Our dense sampling allowed us to describe in detail the population structure, provide insight into genomic history of numerous European and Asian populations, and significantly increase quantity of genetic data available for modern populations in region of North Eurasia. Our study doubles the amount of genome-wide profiles available for this region.

We detected unusually high amount of shared identical-by-descent (IBD) genomic segments between several Siberian populations, such as Khanty and Ket, providing evidence of genetic relatedness across vast geographic distances and between speakers of different language families. Additionally, we observed excessive IBD sharing between Khanty and Bashkir, a group of Turkic speakers from Southern Urals region. While adding some weight to the “Finno-Ugric” origin of Bashkir, our studies highlighted that the Bashkir genepool lacks the main “core”, being a multi-layered amalgamation of Turkic, Ugric, Finnish and Indo-European contributions, which points at intricacy of genetic interface between Turkic and Uralic populations. Comparison of the genetic structure of Siberian ethnicities and the geography of the region they inhabit point at existence of the “Great Siberian Vortex” directing genetic exchanges in populations across the Siberian part of Asia.

Slavic speakers of Eastern Europe are, in general, very similar in their genetic composition. Ukrainians, Belarusians and Russians have almost identical proportions of Caucasus and Northern European components and have virtually no Asian influence. We capitalized on wide geographic span of our sampling to address intriguing question about the place of origin of Russian Starovers, an enigmatic Eastern Orthodox Old Believers religious group relocated to Siberia in seventeenth century. A comparative reAdmix analysis, complemented by IBD sharing, placed their roots in the region of the Northern European Plain, occupied by North Russians and Finno-Ugric Komi and Karelian people. Russians from Novosibirsk and Russian Starover exhibit ancestral proportions close to that of European Eastern Slavs, however, they also include between five to 10 % of Central Siberian ancestry, not present at this level in their European counterparts.

**Conclusions:**

Our project has patched the hole in the genetic map of Eurasia: we demonstrated complexity of genetic structure of Northern Eurasians, existence of East-West and North-South genetic gradients, and assessed different inputs of ancient populations into modern populations.

**Electronic supplementary material:**

The online version of this article (10.1186/s12863-017-0578-3) contains supplementary material, which is available to authorized users.

## Background

The phenotypic diversity of modern humans was shaped under the combined pressure of environment and social relations. Placing the studies of human genetic variation into a geographical context provides powerful insights into how historical events, patterns of migration, and natural selection have led to genetic distinctions between various present-day populations [1, 2]. Moreover, genomic investigations may aid in resolving historic record discrepancies by confirming or rejecting hypotheses of ancient invasions and ethnic intermixing events.

While human genetic diversity has been sampled extensively in many areas of the globe [3–6], a sizeable gap remains in the region of Northern Eurasia (region including Russia and neighboring countries from the former Soviet Union) which spans from the Arctic Ocean down to Inner Asia, and from Eastern Europe to the Pacific Ocean. Though in total, human populations inhabiting this region were analyzed among others in several genome-wide studies [7–22] most of them were focused on other regions and included just a limited number of Northern Eurasian populations. Only five published studies were focused on the areas within North Eurasia: two papers of Yunusbayev [15, 19] investigated genetic composition of the Caucasus and Turkic speaking groups; [18] focused on Balto-Slavic speakers; [20] and [10] studies were even more limited in their geographic coverage. Thus, a panoramic genetic study covering all of Northern Eurasia is still lacking. The Russian Federation represents a unique setting for genetic studies because of its multitude of ethnicities with the evidence for admixture interspersed across several isolated communities. Further, its enormous space and considerable climatic variation created a range of distinct environmental niches which may have contributed to differential shaping of the genomes. However, the limited number of sampled populations in the published datasets translates into significantly less coverage incomparable to that for the western and central regions of Europe.

Here we present high-quality genome-wide analysis of 30 diverse populations from Russia and neighbouring countries (see Table 1). Some of these populations have been previously studied on a smaller scale, and some been sampled here for the first time. Though full genome sequences on population level started to accumulate worldwide extensively, only 246 full genomes were so far published for the Russian populations [10, 11, 16, 17, 23–26]. In contrast, genome-wide genotypes were published for 963 samples from 51 Russian populations [10, 16]. So, the genotyping arrays remain the most important source of genomic variation within Northern Eurasia. Here we double this aggregate dataset by publishing genome-wide genotype data on 1076 samples (1019 of them unrelated) from 30 populations of Russia and adjacent countries.

**Table 1:** Populations genotyped for this study. For each population, the number of unrelated individuals genotyped, type of microarray used, and geographic coordinates are given. The country is Russia unless specified otherwise

Population	Sample size	Platform	Latitude	Longitude
Abhaz	36	Illumina Quad 610	41.5	43.0
Adygei	33	Illumina Quad 610	44.9	39.3
Bashkir Arkhangelskiy district	20	Illumina Quad 610	64.6	40.6
Bashkir Burzyansky district	14	Illumina Quad 610	53.5	56.7
Belarus (Belorussia)	34	Illumina Quad 610	53.2	28.1
Buryat	45	Illumina Quad 370	54.8	112.2
Chechen	35	Illumina Quad 610	43.6	46.1
Cherkes	36	Illumina Quad 610	44.2	42.1
Chinese (China)	13	Illumina Quad 370	31.3	121.5
Chuvash	30	Illumina Quad 610	55.4	47.0
Kabardin	35	Illumina Quad 610	43.2	43.2
Karachay	27	Illumina Quad 610	43.5	41.8
Karelians	35	Illumina Quad 610	63.7	32.7
Kazakh (Kazakhstan)	48	Illumina Quad 370	45.7	69.0
Ket	31	Illumina Quad 610	66.5	84.5
Khanty	29	Illumina Quad 370	62.0	74.8
Komi	32	Illumina Quad 610	64.3	53.8
Kyrgyz (Kyrgyzstan)^a^	35 (22/13)	Illumina Quad 610/Illumina Quad 370	41.6	74.7
Megrel (Georgia)	36	Illumina Quad 610	41.9	42.5
Moldovan	32	Illumina Quad 610	47.2	28.6
Mordva (Moksha & Erzya)	33	Illumina Quad 610	54.3	44.0
Osetin	35	Illumina Quad 610	42.9	44.3
Russian Novosibirsk	39	Illumina Quad 370	55.1	82.9
Russian Starover	41	Illumina Quad 370	57.3	67.9
Tatar	41	Illumina Quad 610	55.2	51.6
Tuva	44	Illumina Quad 370	51.5	95.4
Udmurt	30	Illumina Quad 610	58.0	52.7
Ukrainian (Ukraine)	36	Illumina Quad 610	50.0	32.9
Uzbek (Uzbekistan)	39	Illumina Quad 610	41.7	62.6
Yakut	45	Illumina Quad 370	66.6	116.7
Total	1019			

Note: ^a^Kyrgyz samples were genotyped on Illumina Quad 610 (22 samples) Illumina Quad 370 (13 samples) platforms

Certain unusually diverse areas were given special consideration, such as the Caucasus, where all the major ethnicities, including Abkhaz, Adygei, Chechen, Cherkes, Kabardian, Karachay, Megrel, and Ossetian were profiled. We have also sampled several unique populations, such as the Ket - an isolated, native Siberian people with a distinct language [10] and the Starover Russians, orthodox Old Believers who left western Russia in the seventeenth century and settled in the dense boreal forests of the banks of Volga and the Russian European North, as well as on the southern outskirts of Siberia [27]. Starovers maintain the liturgical and ritual practices of the Russian Orthodox Church as they existed prior to the reforms of Patriarch Nikon of Moscow between 1652 and 1666. In this work, we studied the descendants of Siberian Starovers, who presumably had limited admixture with other groups.

Several independent groups of researchers [1, 2, 8, 28–33] have analyzed the relation between genetic variation and geography, with a variety of biogeographical analysis techniques developed [2, 8, 32–39]. This relationship was extensively studied for European populations [32, 33, 35], for Indian casts [40–42], and, more generally, for world-wide populations [8]. Here we present a detailed analysis of Northern Eurasian populations inhabiting the territories of Russian Federation, and neighbouring countries.

## Methods

### Sample collection and quality controls

DNA samples (*N* = 1076) were collected in course of study expeditions into different parts of Russia, Kazakhstan, Georgia, Uzbekistan and Kyrgyzstan. Samples were genotyped on the Illumina Infinium 370-Duo, 370-Quad, or 610-Quad arrays (https://support.illumina.com/downloads/humancnv370-duo_v10_product_files.html, https://support.illumina.com/downloads/humancnv370-quad_v30_product_files.html, https://support.illumina.com/array/array_kits/human610-quad_beadchip_kit.html). The number of samples per population, source of the samples, and the type of microarray used are given in the Table 1.

All DNA samples were subjected to the following quality control procedures: samples with genotyping success rates <90% were removed, as were male samples with ≥1% heterozygous markers on the *X* chromosome or female samples with ≤20% heterozygous *X* chromosome markers. Across retained samples, 95% cut-off for SNP presence was imposed. (Further details are provided in the Additional file 1: Figure S1).

According to the meta-data from questionnaires, all study volunteers were unrelated to each other. Nevertheless, the dataset was analysed for the presence of cryptic relatedness by calculating the kinship coefficients separately in each ethnic group using the *King* software [43], assuming the presence of population structure. For 29 related pairs of individuals with a threshold of kinship coefficient set at ≥0.177 [43], the sample with the lower genotyping-call rate was excluded from further analysis, thus, only 1019 samples remained out of 1076.

To reduce the effect of missing data, the marker panel was limited to autosomal SNPs with genotyping success rates ≥99.5%. For each set of samples, more than 200,000 markers were analysed.

**Fig. 1:**
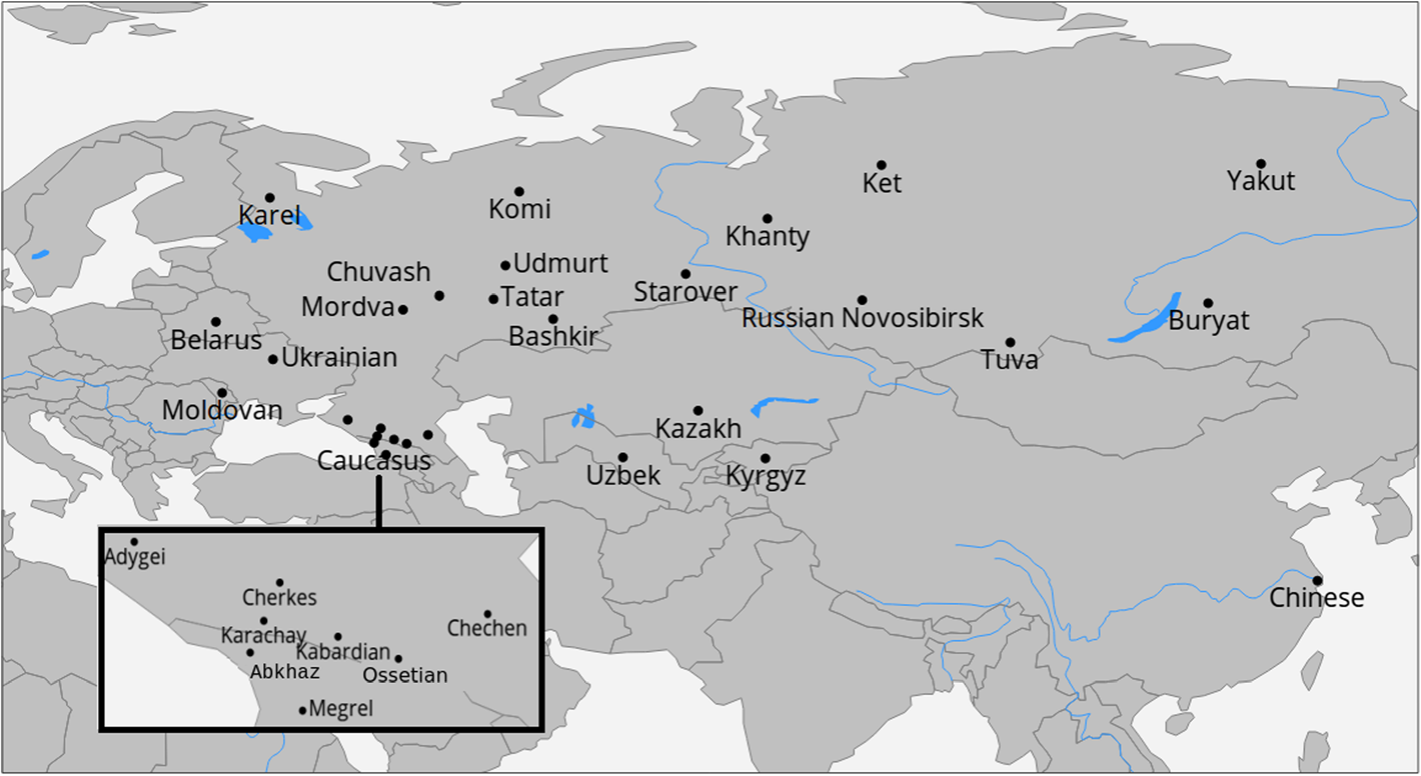
Geographic position of samples in our study

Geographic locations of the sampled populations are presented in the Fig. 1 (samples from this study) or online at http://tinyurl.com/biengi (all samples used for the analysis).

### Origin of the samples

To provide a uniform representation of ethnic diversity in Russia we sampled broadly over the country (see Fig. 1 and Table 1) and adjacent territories. In total, genome-wide variation was accessed in 1019 individuals from five countries across the former Soviet Union. All except one of the studied populations (Chinese) were covered by at least 20 samples (see Table 1). To limit influence of recent admixture, we ensured sampling of villagers who reportedly were settled in the sample place for at least three generations (up to grandparents).

### Datasets

The collected data were assembled in three datasets.

1. The “Extended” dataset includes all individuals genotyped in this study combined with selected previously published modern and ancient samples from Northern Eurasia, which extend geographic span of our study and provide necessary populational context for our analyses (1353 individuals from 55 populations, plus 11 ancient samples, shown in Additional file 3: Table S1a). The “Extended” dataset was used for ADMIXTURE analysis.
2. The “Core” dataset contains samples genotyped in this study (1019 individuals shown in Additional file 3: Table S1b). The “Core” dataset was used to calculate IBD sharing and *f3* statistics.
3. The “Ancient” dataset includes all individuals genotyped in this study combined with European samples from “1000 Genomes” project as well as previously published ancient DNA samples (1232 individuals shown in Additional file 3: Table S1c). This dataset was used to calculate the *f3* outgroup statistics.

### ADMITURE

ADMIXTURE [44, 45] algorithm was used in unsupervised mode to determine the population structure. The number of components (K) was varied from 2 to 10, and cross-validation errors was recorded for all values of K.

For GPS [8] and reAdmix [39] analyses, the reference dataset was obtained from E Elhaik, T Tatarinova, D Chebotarev, IS Piras, C Maria Calò, A De Montis, M Atzori, M Marini, S Tofanelli, P Francalacci, et al. [8]. To enable the comparison with earlier published results, SNPs were converted to the 9-dimensional admixture vectors (“North East Asian”, “Mediterranean”, “South African”, “South West Asian”, “Native American”, Oceanian”, “South East Asian”, “Northern European”, “Sub-Saharan African”) using the ADMIXTURE [44, 45] algorithm in supervised mode.

### IBD calculations

IBD blocks were identified for every pair of individuals in the “Core” dataset using fastIBD algorithm implemented in BEAGLE 3.3 [46]. For further analysis, the amount of shared IBD was calculated separately in two bins: short 1–3 cM blocks and longer 4–10 cM blocks. Total amount of shared IBD was then averaged per pair of individuals.

### *F3* calculations

The three-population statistics were calculated in *threepop* software included in Treemix [47] with the block size parameter (−k) set to 500 SNPs (other parameters set to default values).

### GPS and reAdmix provenance identification

To validate compatibility of the genotyping technologies based on different platforms, we applied GPS [8] and reAdmix [39] to the combined dataset consisting of populations sampled in our study along with ones previously sampled by the National Genographic project, HapMap, and “1000 genomes”. Depending on the database coverage of a selected region and ethnic group, GPS/reAdmix accuracies differ. For example, in this analysis, self-identifications of all profiled Kyrgyz, 75% of Kabardian, and 60% self-reported Ket were correctly identified by GPS. For populations not covered by reference database, locations were triangulated using neighboring ethnic groups.

## Results

### Population structure

**Fig. 2:**
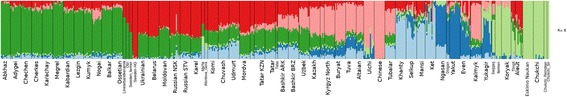
Admixture proportions in studied populations, K = 6. Populations from the Extended dataset. Abbreviated population codes: NSK - Russians from Novosibirsk; STV -Starover Russians; ARK: Bashkirs from Arkhangelskiy district; BRZ - Bashkirs from Burzyansky district

In our study, collection points spanned the latitudes from the Northern Europe to Caucasus and longitudes from Eastern Europe to far shores of Siberia. The results of unsupervised analysis of the “extended” dataset with ADMIXTURE [44, 45] varying the number of ancestral clusters (K) from 2 to 10 (Additional file 2: Figure S2) were most informative for K = 6 (Fig.2), which resulted in lowest cross-validation error and meaningful distribution of components between studied populations.

To enable direct comparison with previous world-wide studies by National Genographic Project [7, 8, 10, 31, 39, 48], ADMIXTURE analysis in supervised mode were performed for K = 9. For each chip type, we selected a subset of SNPs (~30 K and ~60 K for the two chip types) that matched National Genographic Project chip Geno 2.0 [7].

Among the profiled populations, the degrees of ancient admixture varied dramatically. While the populations residing in the Caucasus and East Siberia regions were mostly represented by a single component (dark green and dark blue, correspondingly), the samples from the Volga-Ural region exhibit substantial admixture of European and Asian components (red, dark green, and light blue). Importantly, for each of the populations, the ratio between components was characteristic of that population (Additional files 3: Tables S2 and S3). Moreover, we observed a subdivision of the European genetic component into two clusters, one most prevalent in the Caucasus (dark green) and another with highest frequency in Northern Europe (red). These two European components jointly account for 50% - 90% of admixture vectors in both Turkic and Uralic speakers of Volga-Ural region, while in Finno-Ugric speakers in Northeast Europe and in all Slavic populations these components account for almost 90% of the gene pool.

Slavic speakers – Russians, Ukrainians, and Belarussians - are similar in their genetic composition. Ukrainians and Belarusians have almost identical proportions of the two “European” components and have virtually no “Asian” admixture. Russians from Siberia - both Novosibirsk residents’ and Russian Eastern Orthodox Old Believers’ (Starover) samples - being genetically close to Slavs residing in Europe, also have between five to 10 % of Central Siberian ancestry (light blue).

Asian genetic ancestry of the profiled populations is represented by four components. Two of them, light green (Beringian) and dark blue (East Siberian), are geographically confined to Northeast Asia, while showing only minor impact on populations west of Ural Mountains. On the contrary, East Asian (pink) and Central Siberian (light blue) components are also present in populations to the west of Ural.

In Central Asian Turkic speakers, including Kazakh and Uzbek, East Asian genetic influence is dominant (>35%), while in Bashkir it is detected at somewhat lower levels (~ 20%). Importantly, in Western Turkic speakers, like Chuvash and Volga Tatar, the East Asian component was detected only in low amounts (~ 5%).

The light blue genetic component dominates genetic landscape of populations inhabiting West and Central Siberia: Ugric-speaking Khanty and Mansi, Samoyedic speaking Selkups and linguistically isolated Ket. However, this ancestry component is present not only in Siberia, but also on the western side of Ural Mountains, though at somewhat lower frequencies - 20-30% in Komi (16% on average) and Udmurt (27% on average) who belong to the Permic branch of Uralic languages. Interestingly, similar levels of this ancestry component (16–23%) are also exhibited by Turkic speaking Chuvash (20% on average) and Bashkir (17% on average), while Tatar, who also reside in the Volga region and have related linguistic and cultural profiles, only show at most 15% (10% on average) of this genetic component. Even lower levels of this ancestry component (<5%) were observed in Turkic speakers of Central Asia.

The Beringian component (light green) is confined exclusive to indigenous populations of Eskimo, Chukchi and Koryak. The East Siberian (dark blue) component is represented by Turkic and Samoyedic speakers of Central Siberian plateau: Yakut, Dolgan and Nganasan. This component is also found at moderate frequencies in Mongolic and Turkic speakers in Baikal region and Central Asia (5–15%), and, at low but discernible frequencies (1–5%), in Turkic speakers residing in Volga-Ural region.

### *f3* population test

ADMIXTURE-guided ancestry clustering suggests that, in most of the populations studied, the genetic background is complex, as it includes at least three hypothetical ancestral components: European, East Asian and North Asian (Siberian). Because the ADMIXTURE-guided ancestry clustering cannot be used *in lieu* of the formal test of admixture, three-population test [40] was conducted to confirm proposed admixture events. Surrogate populations were selected for each of three ancestral components, followed by *f3* test of admixture, to find out whether a target population is admixed between two source populations. The combinations of source and target populations and the Z score in the *f3* test are reported in Additional file 3: Table S4.

Admixture scenarios were tested in sampled populations geographically grouped as Northern Europe, Volga-Ural region and Central Asia and, while assuming two-way mixture between European and East Asian, or between European and Central Siberian surrogate populations. Significant Z scores (Z < −3) for admixture between East European (Belarus, Ukrainian) and either East Asian (Tuva, Yakut) or Central Siberian (Khanty, Ket) populations were obtained for all populations of Northern Europe and Volga-Ural region.

Note, that the value of *f3(X; A, B)* (and the corresponding Z score) is negative if X is a mixture of A and B. Among all pairs compared, the most negative *f3* values were obtained for populations of Volga-Ural region using East Slavs and Siberians as source groups (Additional file 3: Table S4). This suggests that both, Western (including ancestors of East Slavs) and Eastern (Siberian) sources of the formation of Volga-Ural populations, which could be also seen from the ADMIXTURE plot (Fig. 2). Although Ukrainians and Belarus received similar scores in the *f3* test, Belarus turned out to be a slightly better proxy for East European component in populations of Volga-Ural region.

Populations from Central Asia also received significant negative *Z* scores in the *f3* test using East Asia and Europe populations as a source, although their best supported surrogate populations were different. For Uzbek and Kazakh, the best surrogate for a European source was Abkhaz, while for Volga-Ural populations it was either Belarus or Ukrainian. For Uzbek, the best East Asian surrogate was Yakut, while for Kazakh and Kyrgyz it was Tuva (followed by Yakut).

### Analysis of IBD blocks

**Fig. 3:**
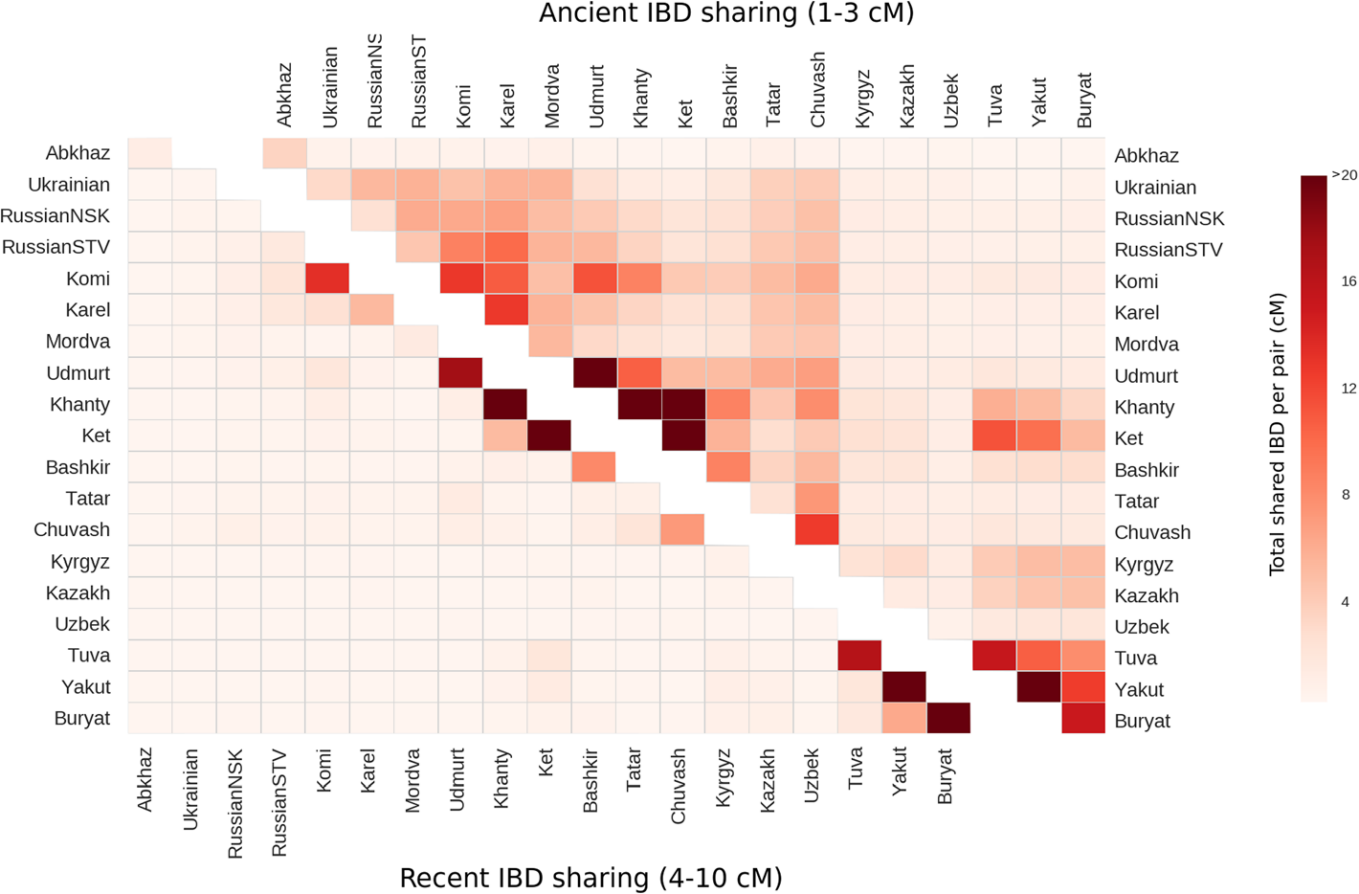
Sharing of ancient and recent IBD blocks between populations in focus regions. IBD sharing is calculated as a sum of IBD segments averaged per pair of individuals. Sharing of IBD blocks is calculated separately for short, ancient blocks (1–3 cM) and more recent 4–10 cM blocks. Recent IBD blocks are typically shared inside the populations, while sharing of ancient IBD blocks is more complex. Darker colors correspond to higher amounts of shared IBD

To identify shared identical-by-descent (IBD) blocks, we used the fastIBD algorithm implemented in the Beagle package [46]. We first calculated total amount of IBD in centimorgans (cM) shared between the populations, which was then averaged per pair of individuals (Additional file 3: Table S6). Since the length of IBD blocks is anti-correlated with their respective age, analysing the distribution of length of blocks allows us to examine patterns in ancestry sharing with temporal resolution [49]. We analysed the amount of shared IBD in two length bins: 1–3 cM (ancient blocks) and 4–10 cM (recent blocks) (Fig. 3 and Additional file 3: Table S5). We focused on three regions, providing the densest sample coverage: Caucasus, Volga basin and Siberia. Populations from the Caucasus share most of IBD blocks between themselves and the amount of shared IBD ranges between 3.26 cM to 12.39 cM per pair, which is roughly comparable to the amount of IBD blocks shared between Eastern European populations in our dataset (Additional file 3: Table S5). A conspicuous exception are the Chechens, who share almost all detected IBD blocks among themselves and only a scant amount of IBD with neighbouring Caucasus populations. This may have happened because the Chechen sample is the only representative of the North-East Caucasus in our dataset. Low amount of IBD shared outside the cluster of Caucasian populations suggests a lack of recent ancestry links with Uralic, Slavic or Turkic people (except for Turkic from the Caucasus) present in our dataset.

The Volga-Ural region is populated by three major language and cultural groups: Uralic, Turkic and Slavic speakers. Bashkir and Tatar are major Turkic groups in the region. Although both ethnic groups live in the same region and their languages are mutually intelligible, we surprisingly detected only a limited amount of ancient IBD blocks shared between them, and their overall IBD sharing pattern is different: Tatar share moderate amount of IBD (3.55–7.35 cM per pair) with all neighbouring populations, while Bashkir share most of their ancient blocks (on average 8.62 cM per pair) with Khanty, a group of Uralic speakers from Western Siberia. We speculate that this disparity between cultural and genetic affinities of Tatar and Bashkir can be attributed to a phenomenon of cultural dominance: the population ancestral to Bashkir adopted the Turkic language during Turkic expansion from the east (language replacement event).

European and Siberian Uralic speakers are separated by the Ural Mountain range. This separation affects sharing of recent IBD blocks: Komi and Udmurt share in the interval of 4–10 cM almost double the amount of IBD they share with Khanty. Interestingly, the situation is different when we look at sharing of ancient IBD blocks: the amount of ancient IBD blocks that Udmurt and Komi share with Khanty (10.63 and 8.62 cM per pair, respectively) is comparable to the amount of ancient IBD shared between them (11.38 cM per pair). These data agree well with the ethnic history of European and Asian Uralic speakers [50]. Udmurt and Komi belong to the Permic branch of the Uralic language family and share a recent origin, as demonstrated by common short IBD blocks, their split from their ancestral Uralic population is dated back to the first half of the 1st millennium BC. Split between ancestors of Asian Uralic people, represented in this paper by Khanty, and ancestors of modern European Uralic ethnic groups (Udmurt and Komi) is much older and dated back approximately to 3rd millennium BC. All modern Uralic populations share common genetic substrate inherited from some ancient Uralic people, reflected in long and similar size ancient IBD blocks shared between Udmurt, Komi and Khanty. All analysed native Siberian populations exhibit high levels of intrapopulation sharing of IBD (Fig 3), which is in line with observed long runs of homozygosity in these populations. High rates of shared IBD blocks were detected also between pairs of Siberian populations, particularly between Ket and Khanty*.*

Following B Yunusbayev, M Metspalu, E Metspalu, A Valeev, S Litvinov, R Valiev, V Akhmetova, E Balanovska, O Balanovsky, S Turdikulova, et al. [19], who observed that the number of shared IBD blocks decline exponentially with the distance between populations, we have calculated linear regression between geographic distance and logarithm of IBD for all pairs of populations from the “Core” dataset (see Additional file 3: Tables S7 and S8). This resulted in the following equation: *log*_10_ *IBD* = 1.772 − 0.0005975 × (*Distance in kilometers*), adjusted *R* ^*2*^ = 0.4923, *p*-value <10^−16^.

**Fig. 4:**
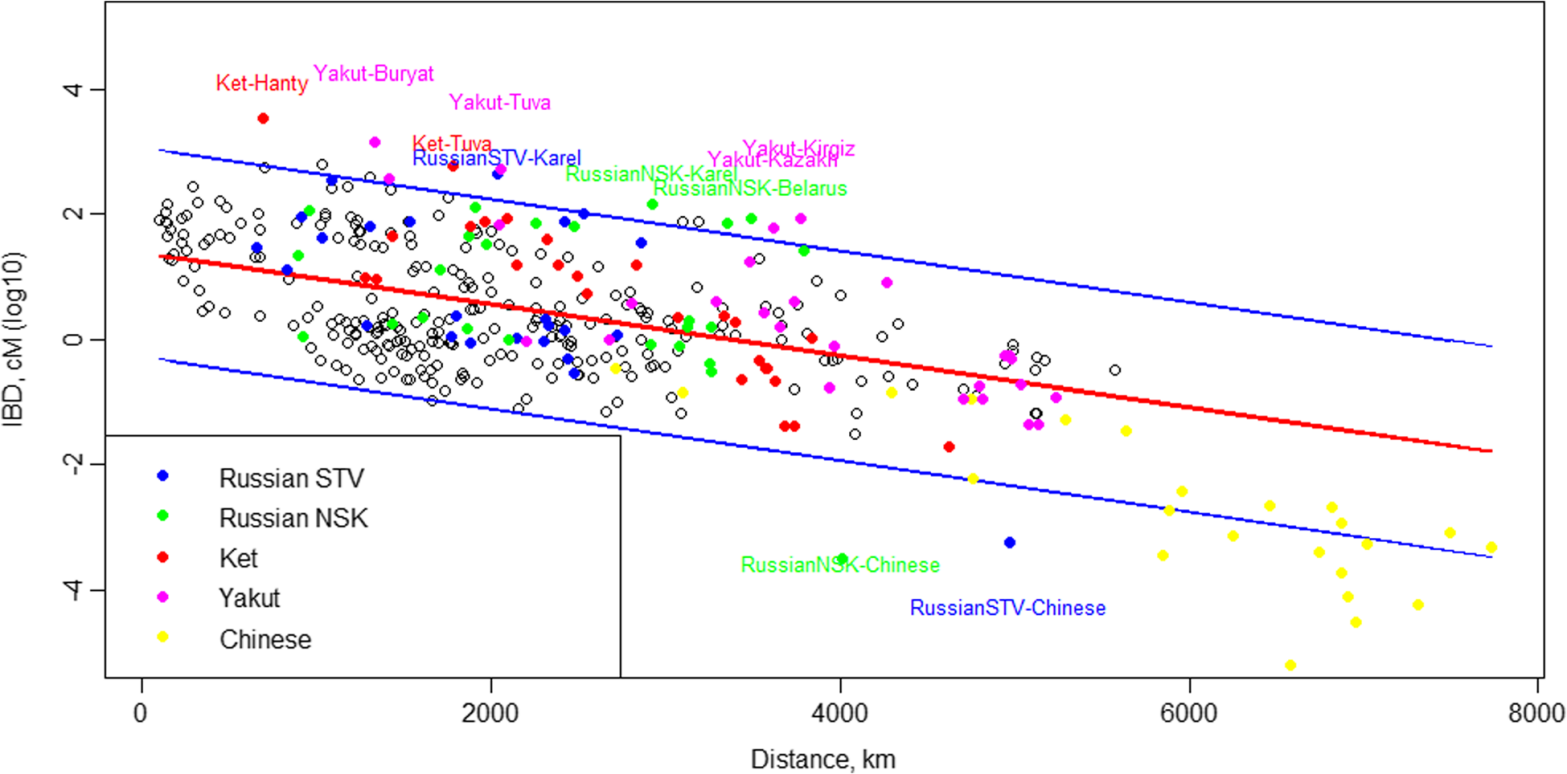
Regression between logarithm of IBD (logIBD) and geographic distance between all pairs of studied Eurasian populations. Red line denotes the regression line and blue lines correspond to 95% prediction interval

Although Russian Starovers appeared in Siberia only about 300 years ago, details of their geographic origin are not clear. Starovers share roughly twice as much of ancient blocks with Komi and Karelian than between themselves, or with other Slavic speaking groups (Additional file 3: Table S5). This finding, corroborated by results of GPS and reAdmix (described below), strongly points to Northern European ancestral ties of Russian Starovers. The most pronounced difference between predicted and observed IBD rate was found for Siberian Russians, the residents of Novosibirsk and the Starovers, and for the Chinese outgroup (Additional file 3: Tables S7 and S8, Fig. 4). This was expected due to relatively recent relocation of Eastern European Slavic individuals to Siberia. Interestingly, one of the Caucasian ethnicities, the Chechens, also share fewer IBD blocks with Chinese than would be expected from the geographical distance separating these populations. This outlier could be possibly explained by underestimation of the degree of apparent geographical isolation of Chechens, occupying hard to reach highlands of Caucasus range, since elevation is not considered by the regression. From patterns of IBD-sharing and from ADMIXTURE-based analysis we see that Caucasus populations differ from their neighbors in the European part of the Russian Federation. One of the factors being that some Caucasus tribes reside at high altitudes of 2000 m above sea level or more, where they have been genetically isolated for centuries [51–54].

**Fig. 5:**
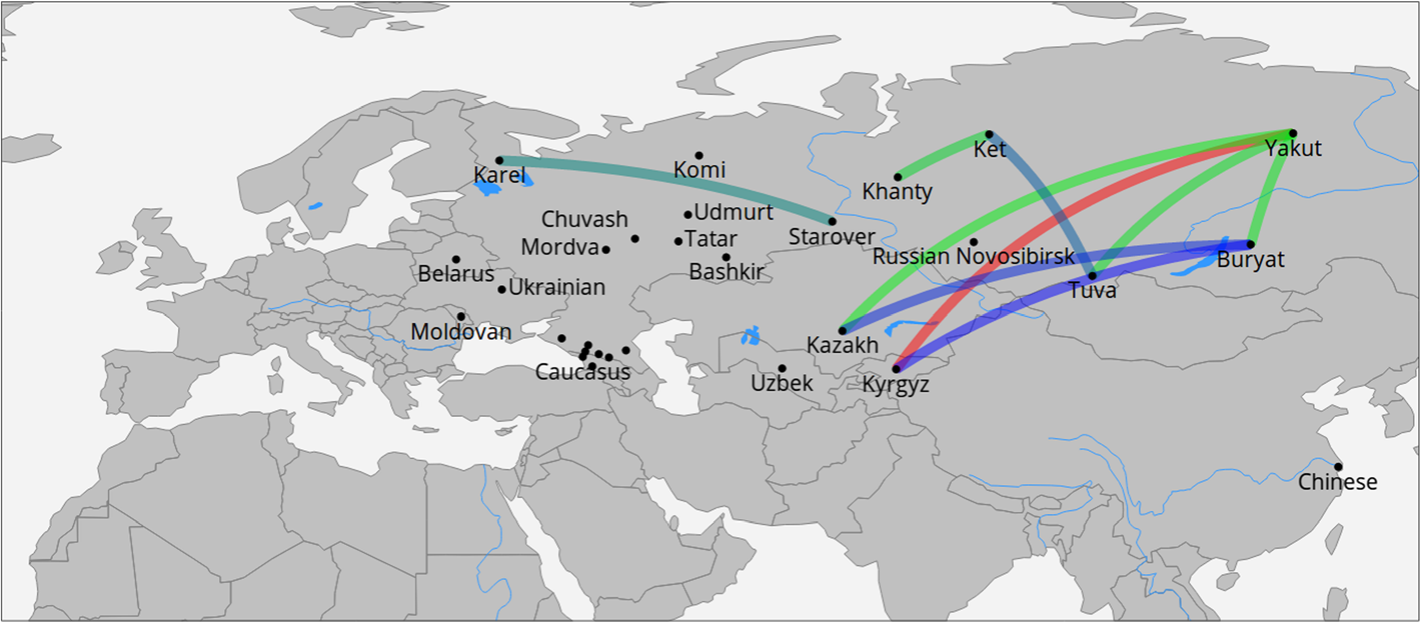
Departures from the expected IBD. Shown populations exceed the expected IBD sharing by more than two standard deviations. Departure from expected values is most pronounced among Siberian populations, and between Karel and Russian Starovers

At the next stage of analysis, Novosibirsk Russian and Chinese populations were excluded, leaving us with 325 population pairs. This analysis produced following equation: *log*_10_ *IBD* = 1.394 − 0.0004121 × (*Distance in kilometers*), adjusted *R* ^*2*^ = 0.2774, p–value <10^−16^. Using this equation, we have identified pairs of populations (using the “core” dataset) that were more than two standard deviations away from the predicted *log*_*10*_ *IBD* (Fig. 4). In this analysis, the departure from the regression line suggests unusual gene flow events, unaccounted for by the calculation of geographic proximity. Most significant departures were observed for Yakut with Kyrgyz, Kazakh, Buryat and Tuva, as well as for Ket with Khanty and Tuva combinations. Starover, who recently migrated to Siberia, again show genetic proximity to Karelian (Fig. 5).

### GPS and reAdmix analyses of self-identified Russians

In our dataset, ethnic Russians constituted the largest population (*N* = 80). These samples came from two groups: residents of Novosibirsk district (39 samples, designated “NSK”) and the Siberian Starover (41 samples, designated “STV”). The Novosibirsk residents were thoroughly surveyed about their ancestors and selected only if reporting at least three preceding Russian generations, while members of the Starover cohort were all assumed to be “authentic” Russians. Since their resettlement from the European part of Russia in the seventeenth century, Russian Starovers deliberately adhered to a strict religious routine and avoided contact with neighbouring Native Siberian populations. Both the Starovers and most Novosibirsk residents are informally considered as “canonical Russians”. Nevertheless, only the Novosibirsk group represents a uniform sample from the modern Russian gene pool.

For all populations sampled in this study, SNP array data were compared to the worldwide collection of populations using Geno 2.0130 K ancestry-informative markers (AIMs) [55]. Both SNP platforms used in our analysis contain a subset of these markers. Chip 370 includes 60,730 AIMs, while chip 610 includes 90,231 AIMs. As demonstrated earlier [8], even in case if admixture vectors are determined with as little as 40,000 AIMs, the difference between the admixture vectors obtained from the complete set of AIMs and the reduced set does not exceed 3%. Therefore, reduction of AIMs to 60,000 or more resides within the range of natural variation and does not affect the accuracy of population assignment.

In the following analysis, we used admixture vectors obtained by ADMIXTURE software run with reference dataset from E Elhaik, T Tatarinova, D Chebotarev, IS Piras, C Maria Calò, A De Montis, M Atzori, M Marini, S Tofanelli, P Francalacci, et al. [8] in supervised mode (К = 9). When admixture vectors for Novosibirsk and Starover Russians were compared, relative weights of the “Northern European” component were found to differ by 2% (t-test *p*-value <0.009). The provenances of the samples were inferred by two algorithms, GPS [8] and reAdmix [39]. For each tested individual, GPS algorithm determines a location on a world map, where people with similar genotypes are most likely to reside. Notably, this algorithm is not suitable for analysis of recently mixed individuals, such as children of parents from two different ethnic groups. When subjected to GPS, a recently admixed sample would result in a report of high uncertainty of prediction.

**Fig. 6:**
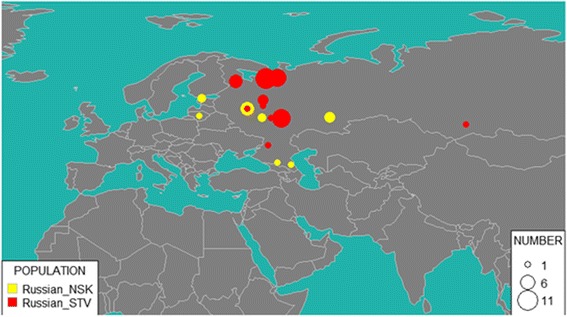
GPS results for NSK (Novosibirsk Russians) and STV (Starover Russians). Size of the bubble corresponds to the number of individuals attributed to the region

To analyze modern Russians from Novosibirsk and Starover Russians, we used Russian diversity panel data genotyped on Geno 2.0 chip (Balanovsky et al., unpublished data). Nearly 37% of self-identified Russians from Novosibirsk were mapped to various Russian populations from European part of Russia: 13% to Tver region, 13% to Arkhangelsk region, 5% to Ryazan region and 3% to Don Cossacks and Vologda region each. Not surprisingly, as many as 27% of Novosibirsk residents were identified as Mordva: 24% as Erzya and 3% as Moksha (Fig. 6). These two subethnic groups were followed by Chuvash (16%), Karelian and Evenki (5% each). Many singular representations of other ethnic groups of the Russian Federation were also reported.

Starover Russians appear to be more closely related to European Russians than Russians in Novosibirsk, with 58% identified as descendants of the migrants from various cities and villages in European part of Russia: for 45% of them, the provenance was traced to Arkhangelsk region, for 7% to Vologda region, for 2% to Yaroslavl region, and for 2% each to Tver region and Don Cossacks. Other notable ethnic component groups include 23% of Erzya and Moksha Mordva collectively, 12% of Karelian, and 5% of Veps.

Altogether, GPS analysis of Starovers suggests that most of them came from northern areas of European Russia. This agrees with the slightly higher value of the Northern European component in Starovers as compared to Novosibirsk Russians.

In addition to the proposed population and geographic location, the GPS algorithm also reports prediction uncertainty calculated from the distance to the nearest reference population. One of the Starover individuals was identified by GPS as a Khakas, a Turkic ethnicity living in the Republic of Khakassia located in southern Siberia, Russia. The same individual had the largest prediction uncertainty (7%) as compared to the average 3% prediction uncertainty for other Starovers samples. Typically, the prediction uncertainties which exceed 4% indicate mixed origin of an individual. For these cases, GPS algorithm should not be used.

**Fig. 7:**
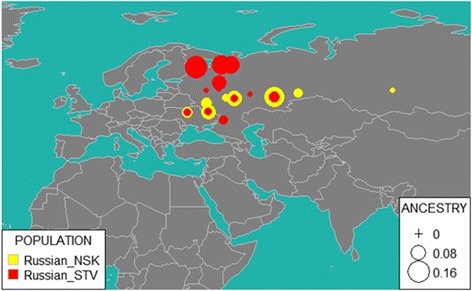
reAdmix for NSK (Novosibirsk Russians) and STV (Starover Russians). Size of the bubble corresponds to average ancestry percentage in a corresponding population

Therefore, for further analysis of Starovers and Novosibirsk individuals, we used reAdmix [39], which represents each individual as weighted sums of modern reference populations (see Fig. 7). In agreement with the GPS results, self-identified Russians from Novosibirsk appear to be more admixed than the Starovers. In Novosibirsk, 37% of genetic input came from ethnic Russians (15% from Northern Russia and 23% from Southern Russia), 25% Finno-Ugric (Veps, Karelian, Mordva), and 38% to other (Buriat, Chukchi, Chuvash, Dolgan, Evenki, Ket, Nenets, Nganasan, Selkup, Tatar, Tuvinian, Yakut, Yukaghir). Among the Starovers, 50% of ancestry was attributed to Russians (with 41% from Northern Russian and 9% of Southern Russia), 33% to Finno-Ugric (Veps, Karelian, Mordva), and 17% to other, including native Siberian populations (such as Tuva, Buryat, Yakut, Ket, Khanty). This observation supports the notion that Siberian Starovers represent relatively large heterogeneous group, which did not stay entirely isolated.

Since the strict religious rules prevented Russian Starovers from marrying members of other ethnic groups, they are commonly believed to be less admixed with native Siberians than other Russian communities in the region. However, our ADMIXTURE analysis showed that the admixture profiles of Russian Starovers and Russians from Novosibirsk are similar (Fig. 2) and that both groups experienced comparable gene flow.

This genetic input can be attributed to multiple known and unknown events in the history of Starovers. We can summarize our observations as follows. According to both GPS and reAdmix analyses, Starover Russians have more significant input from Northern Russians and Finno-Ugric populations than from the South of Russia. Novosibirsk Russians represent a typical mixed Russian population of the early twenty-first century; lesser degree of admixture in the genomes of Starovers point at an increase in the rates of admixture of Russian populations with neighboring ethnicities that occurred in the last 300–400 years.

### *f3* outgroup analysis of relatedness to ancient genomes

Earlier comparative studies of ancient and modern human DNA have helped to delineate human migration routes around the world [56–60]. We used *f3* outgroup statistics [61] to test for shared genetic drift between our studied populations and selected ancient populations, namely East European hunter gatherers, Caucasus hunter gatherers, Anatolian farmers and Mal’ta (See Additional file 3: Table S1c). It was demonstrated that *f3* is positive if and only if the branch supporting the population tree is longer than the two branches discordant with the population tree [62]. Therefore, large positive values of *f3* show that the two tested populations had a large amount of shared population drift.

**Fig. 8:**
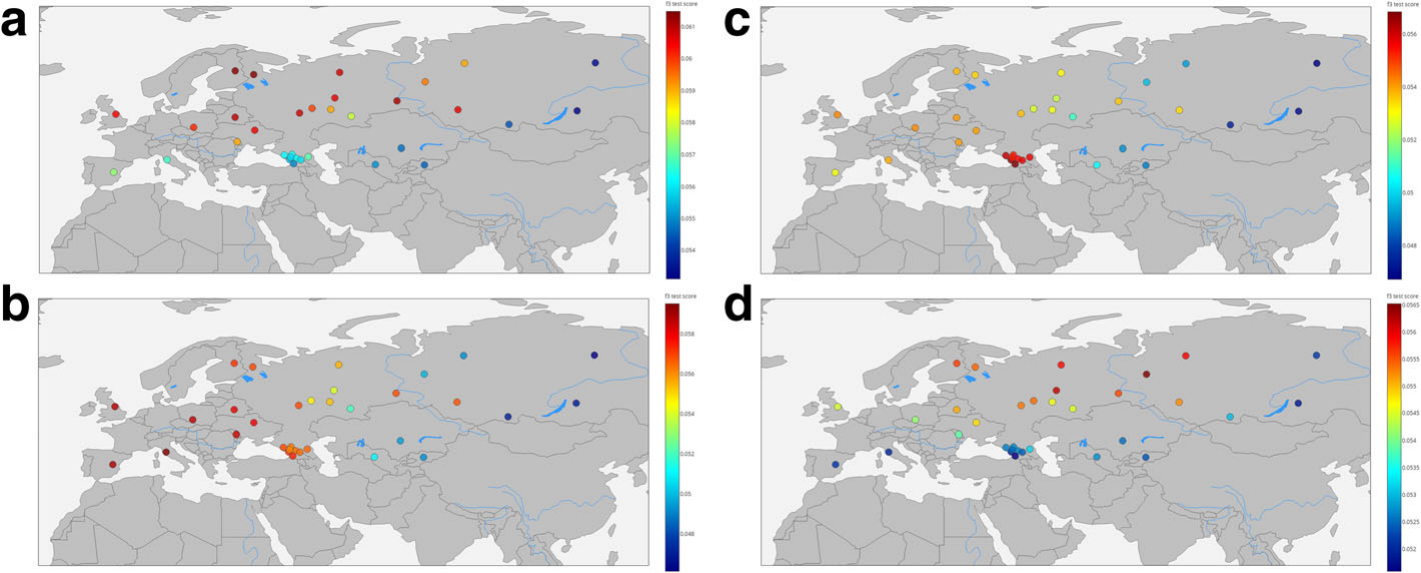
*f3* values to estimate (**a**) Eastern European Hunter-Gatherer, **b** Neolithic Farmer, **c** Caucasus hunter-gatherer, and **d**) Mal’ta (Ancient North Eurasian) ancestry in modern humans

All populations from the “Extended” dataset were used as test populations, with Mal’ta [9], Eastern European hunter-gatherers [56, 58], Caucasus hunter-gatherers (Jones et al. 2015) and Neolithic samples (Mathieson et al. 2015) as the reference and Yoruba as an outgroup (Additional file 3: Table S9). The summary of the findings is shown in the Fig. 8.

This analysis confirms local inheritance of genetic structure between ancient and modern populations, as evident from consideration of aDNA samples from the Caucasus and Europe. We did not find the “source” population for our Eastern Siberian samples (Yakut and Buryat). We also confirmed that modern European population is an amalgamation of ancient European Hunter-Gatherers with Neolithic Farmers. [57, 63–65]. Neolithic Farmers’ genetic influence is present in a wide range of modern Eurasian populations (from the Iberian Peninsula in the West to the Altay mountains in the East). East of Altay the signal fades. Genetic signal from European Hunter Gatherers is present across several Northern Eurasian populations. The modern populations of the Caucasus show a strong signal from Caucasus hunter gatherers, that is almost absent elsewhere. Ancient North Europeans (represented by Mal’ta boy) left their genetic mark on several genomes of modern Northern Eurasians, without affecting Western or Southern Europeans or Eastern Siberians or Central Asians.

## Discussion

Since the pioneering effort by the HapMap Consortium made in 2003 [4], multiple studies were conducted to investigate human genetic diversity, population structure, migration routes, and genotype-phenotype association [2, 8, 16, 32, 33, 55, 66–75]. These studies produced a variety of computational tools and reference datasets, leaving just a few blind spots.

One of these blind spots is in Russia, where only a handful of genome-wide human variation studies were conducted to date [10, 16, 17, 19, 23]. In this work, using the whole-genome SNP analysis, we surveyed 1019 individuals from Northern Eurasia for their genetic diversity. Newly acquired genome-wide high-density coverage for almost 30 ethnic groups in Russia enabled us to perform both inter-population and population-specific analyses. Combined with genome sequencing data available for a limited number of individuals, such as described in [23], our study provides one of the most comprehensive datasets covering genetic variation in Russia.

The relationship between genetics and geography was analysed by a combination of ADMIXTURE-based and IBD sharing approaches. We showed that Russian populations of diverse demographic histories and geographic localization share many genetic features, as reflected in their relatively tight ADMIXTURE groupings and outputs of GPS and reAdmix. The apparent positioning of some Russian samples in the genetic space of Caucasus and Siberian populations may reflect either traces of historical assimilation of these groups during the expansion of the Russians, or a recent contribution from neighbouring ethnic groups to the genomes of specific individuals. When we compared Starover and Novosibirsk Russians, representing snapshots of historical (as old as the seventeenth century) and modern (twenty-first century) Russian population, respectively, an apparent recent increase in the rates of admixture with various neighbouring population was evident. Admixture profiles of Modern Novosibirsk Russians have a lower percentage of Northern European components compared to Starovers Russians. In addition, various analyses including GPS, IBD and reAdmix suggest that Starover Russians were genetically influenced by Finno-Ugric people; this hypothesis agrees with the historical record concerning the patterns of Starover migrations within Russian Empire.

One of most curious findings involved the Bashkir, an ethnicity with an extremely complex historical background. There are three main theories describing Bashkir origins: “Turkic”, “Finno-Ugric”, and “Iranian” [76, 77]. According to the “Turkic” theory, most Bashkir genetic ancestry was formed by Turkic tribes migrating from Central Asia in the first millennium AD. The “Finno-Ugric” theory stipulates that the nucleus of Bashkir ancestry was formed by the Magyar (Hungarians), who were later assimilated by Turkic tribes and adopted a Turkic language, while the “Iranian” theory considers Bashkir to be descendants of Sarmatians from the southern Ural.

Speaking generally, our findings add weight to “Finno-Ugric” theory of the origin of Bashkir. A majority of Bashkir IBD fragments were shared with Khanty, an ethnicity related to Magyar. Interestingly, some works point out that before the thirteenth century the Hungarians were commonly called Bashkir ([78], pp. 289–294). It is surmised that the Magyar ethnicity was formed in the region between Volga and the Ural Mountains, then, at the end of the sixth century AD, moved to the Don-Kuban steppes abandoned by the Proto-Bulgarians followed by the move to their present location between Dnieper and Danube somewhat later.

Further analyses (ADMIXTURE and recent IBD) pointed to proximity of Bashkir to Turkic-speaking Tatar and Chuvash as well as to Finno-Ugric Udmurt and Khanty. In addition, results of *f3* outgroup analysis indicate that Bashkir, in contrary to other Turkic speakers, were strongly influenced by Ancient Northern Eurasians, highlighting a mismatch of their cultural background and genetic ancestry and an intricacy of the historic interface between Turkic and Uralic populations. As a general pattern, the Eastern European speakers of Uralic languages share large amounts of IBD with Khanty and Ket, with Turkic speaking Bashkir being added to this rule.

It is noteworthy that the genomes of closest linguistic relatives of Bashkir, Volga Tatar, bears very little traces of East Asian or Central Siberian ancestry. Volga Tatar are a mix between Bulgar who carried a large Finno-Ugric component, Pecheneg, Kuman, Khazar, local Finno-Ugric tribes, and even Alan. Therefore, Volga Tatars are predominantly European ethnicity with a tiny contribution of East-Asian component. As most Tatar’ IBD is shared with various Turkic and Uralic populations from Volga-Ural region, an amalgamation of various cultures is evident. When the original Finno-Ugric speaking people were conquered by Turkic tribes, both Tatar and Chuvash are likely to have experience language replacement, while retaining their genetic core. Most likely, these events took place sometime around VIII century AD, after the relocation of Bulgar tribes to Volga and Kama river basins, and expansion of Turkic people.

We speculate that Bashkir, Tatar, Chuvash and Finno-Ugric speakers from Volga basin has a common Turkic component, which could have been acquired as a result of Turkic expansion to Volga-Urals region. However, the original Finno-Ugric substrate was not homogeneous: Tatar and Chuvash genomes carry mainly “Finno-Permic” component, while Bashkir carry the “Magyar” one. The fraction of the Turkic component in Bashkir is, undoubtedly, quite significant, and larger than that in Tatar and Chuvash. This component reflects the South Siberian influence on Bashkir, which makes them related to Altai, Kyrgyz, Tuvinian, and Kazakh people.

As a standalone approach, an analysis of shared IBD is not sufficient to support the Finno-Ugric hypothesis of Bashkir origin as a sole source, while pointing at temporal separation of genetic components in Bashkir. Hence, we demonstrated that Bashkir genepool is a multifaceted, multicomponent system, lacking the main “core”; it is an amalgamation of Turkic, Ugric, Finnish and Indo-European contributions. In this mosaic, it is impossible to identify the leading element. Therefore, Bashkir are the most genetically diverse ethnic group of the Volga-Urals region.

Many Siberian populations share an unusually high amount of IBD, which may be explained by a combination of the following factors: 1) shared origin, 2) relative isolation from outside world, 3) rapid recent population growth and strong founder effect in Yakut, Buryat, and Tuva, or 4) gene flow facilitated by some migrating population. The structure of these population also reflects the role of multiple South-North travel routes along the great waterways of Ob, Yenisei, and Lena, while the Siberian taiga, which is notoriously hard to traverse, to some degree prevented lateral access. On the other hand, Southern Siberia, where the steppes border the forests, is easier to travel. The same is true for the Northern Siberia, where the cold, flat tundra is suitable for travel by deer herders. These geographical limitations corralled the East-West migration to either “northern” or “southern” corridors and North-South migrations to the banks of great Siberian rivers. The footprints of these geographical restrictions could be seen in the patterns of IBD sharing between the Siberian populations studied. We christened it as the “Siberian genetic vortex”.

High IBD between West Siberian Ket and Khanty populations may reflect their relatively recent admixture with Selkup. Close genetic relationships between Ket and Tuvan can be explained by the existence of an ancient pre-Turkic and pre-Samoyedic Yenisei substrate which constitutes the main genetic component in Ket and still present in Tuva due to assimilation of extinct Yeniseian peoples (such as Kott, Arin, and Pumpokol) [79] inhabited Yenisei source area in the Southern Siberia [80].

High levels of shared IBD blocks in Altaic-speaking populations from Southern Siberia (Tuva, Buryat), North Asia (Yakut) and Central Asia (Kyrgyz) supports their recently formed common genetic core, which is geographically related to the Altay-Sayan Mountains region in Southern Siberia. Yakut and Kyrgyz populations which are now distant from this region were resettled from Southern Siberia relatively recently. It is accepted that ancestors of Yakuts (Kurykan) migrated from the Southern Yenisei to Lake Baikal area in seventh century AD, and then travelled the Lena river North in 12th -14th centuries AD [81], while Kyrgyz, who until recently were known as Yenisei Kyrgyz, migrated from Southern Siberia to Central Asia in 13th - 15th centuries AD after the collapse of the Mongol Empire [82].

The discovery of long runs of homozygosity in native Siberian populations (such as Tuva, Buryat, Yakut, Ket, Khanty) supports the earlier finding of pronounced founder effects and low genetic diversity in Siberians due to genetic drift, isolation by distance and recent population expansion events, that were made using the Y-chromosome analysis [83–89].

Comparative analysis of modern and ancient genomes suggests that Western Siberians have more Ancient North European ancestry (represented by Mal’ta) than other populations of the Russian Federation. Other studied populations show genetic affinity to various ancient genomes, either co-located with modern inhabitants, pointing to direct gene flow and relatively sessile population, or geographically removed, pointing to their migration to currently occupied locations.

We see that the shared genetic drift associated with hunter gatherers (Fig. 8) is correlated with Northern European ancestry of studied individuals. At the same time, the shared genetic drift of farmers has a pronounced gradient: it is large in the areas suitable for agriculture and drops to zero in Ket and Khanty-inhabited boreal forest areas of Siberia, where the climate is harsh and summers are too short for a sustainable harvest. In Siberian forests, the signal of Neolithic ancestry is no longer detected, but the ancient northern Eurasian (ANE) signal predominates instead. Possibly, the ancient Northern Eurasians met with more western groups of ancient hunters or with ancient farmers in the steppe, formed a certain population resembling the steppe samples of Yamnaya and Afanasyevo cultures, which then spread this North Eurasian component across and beyond the boreal forests of Siberia. This suggests an extensive westward migration from the steppe, discussed in detail elsewhere [56]. It is also possible that there was wave of northern or western Europeans migrating to the steppes from an opposite direction.

## Conclusions

Our project has filled an important lacuna in the genetic map of Eurasia. We revealed the complexity of genetic structure of Northern Eurasians, the existence of East-West and North-South genetic gradients, and varying inputs of ancient populations into modern populations. In particular, we have collected evidence in support of Finno-Ugric influence on the formation of Bashkir, shed light onto the genetic make-up of Russian Starovers (Old Believers), and postulated the existence of a Great Siberian Vortex directing genetic exchanges in populations across the Siberian part of Asia.

## Additional files

Additional file 1: Figure S1. Quality control process. (PDF 25 kb)

Additional file 2: Figure S2. Results of ADMIXTURE for K = 2–10. (PNG 157 kb)

Additional file 3: Table S1a. List of samples included in “Extended” dataset. **Table S1b** List of samples included in “Core” dataset. **Table S1c** List of samples included in “Ancient” dataset. **Table S2** Results of ADMIXTURE for K = 9. **Table S3** Results of ADMIXTURE for K = 6, 7, 8. **Table S4** Results of f3 test. **Table S5** Results of IBD sharing analysis in 1–3 cM and 4–10 cM bins. **Table S6** Total amount of shared IBD between populations. **Table S7** Standard residue of linear regression analysis of distance-IBD sharing. **Table S8** Distance and shared IBD between pairs of populations. **Table S9**: Results of f3 outgroup test with ancient samples. (XLSX 482 kb)
