# Supplementary figures and images for: Between Lake Baikal and the Baltic Sea: genomic history of the gateway to Europe
Source: BMC Genet. 2017 Dec 28;18(Suppl 1):110. doi: 10.1186/s12863-017-0578-3 (PMC5751809; doi:10.1186/s12863-017-0578-3)

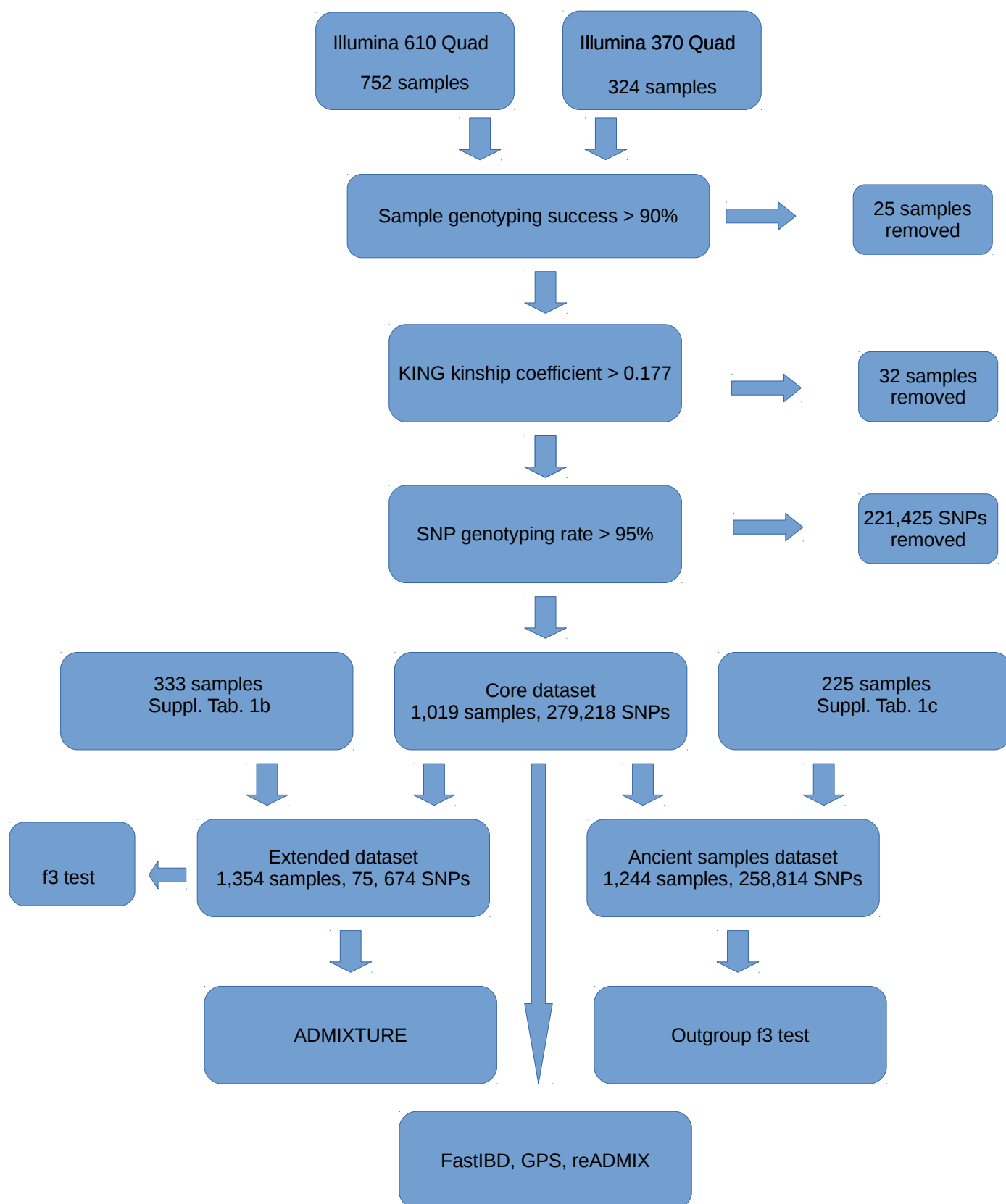

Supplement: Supplementary file 1 — Quality control process. (PDF 25 kb) [file 12863_2017_578_MOESM1_ESM.pdf]

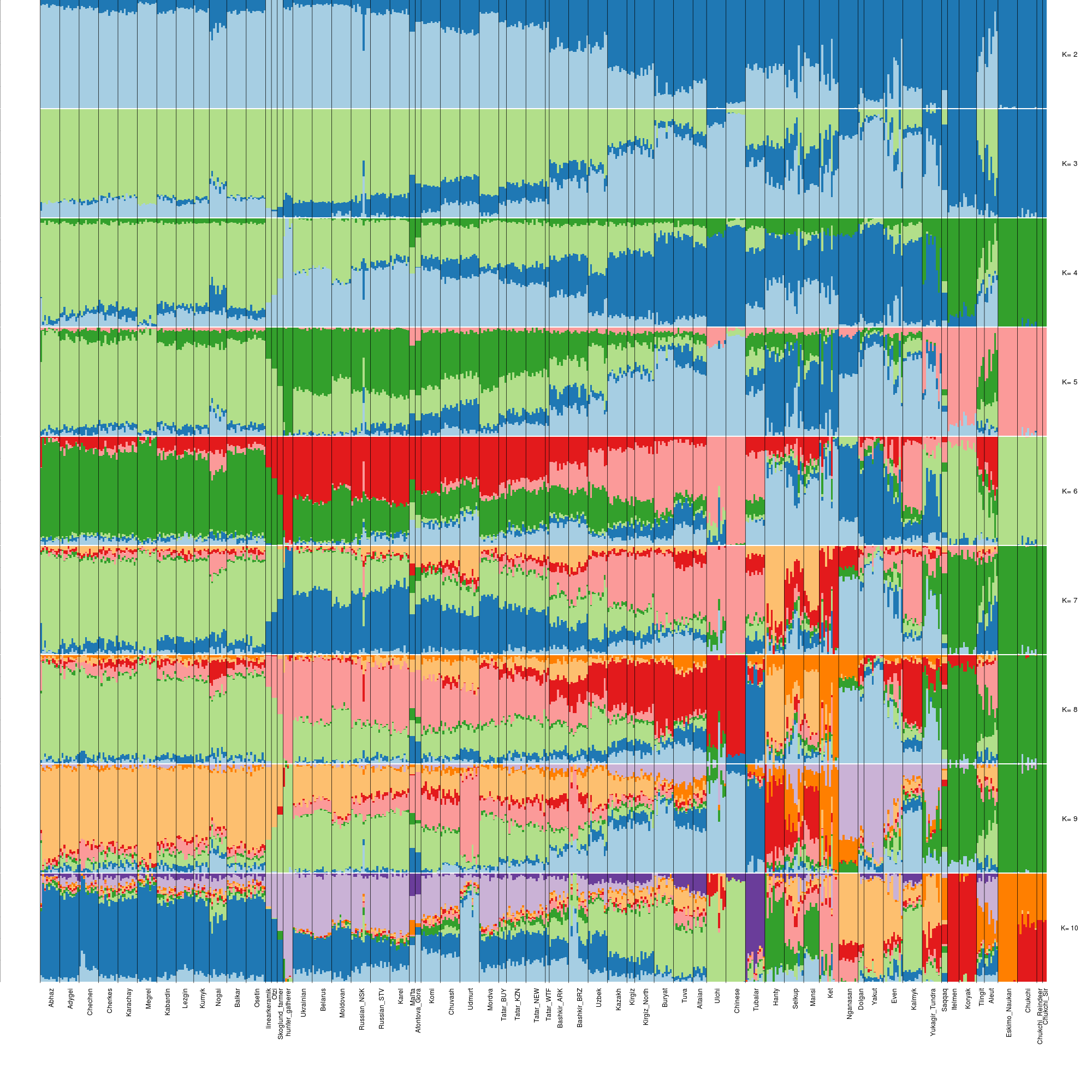

Supplement: Supplementary file 2 — Results of ADMIXTURE for K = 2–10. (PNG 157 kb) [file 12863_2017_578_MOESM2_ESM.png]
